# A polymorphic variant of the insulin-like growth factor 1 (IGF-1) receptor correlates with male longevity in the Italian population: a genetic study and evaluation of circulating IGF-1 from the "Treviso Longeva (TRELONG)" study

**DOI:** 10.1186/1471-2318-9-19

**Published:** 2009-05-21

**Authors:** Diego Albani, Sara Batelli, Letizia Polito, Angelica Vittori, Marzia Pesaresi, Giovanni Battista Gajo, Sergio De Angeli, Andrea Zanardo, Maurizio Gallucci, Gianluigi Forloni

**Affiliations:** 1 Department of Neuroscience, "Mario Negri" Institute for Pharmacological Research, via La Masa 19, I-20156 Milan, Italy; 2 ARGEI, Interdisciplinary Geriatric Research Association, Trento Trieste Avenue 19, I-31100 Treviso, Italy; 3 Trasfusional Center Treviso General Hospital, Piazza Ospedale 1, I-31100 Treviso, Italy; 4 Clinical Chemistry Laboratory Treviso General Hospital, Piazza Ospedale, 1 I-31100 Treviso Italy; 5 Treviso General Hospital, Piazza Ospedale, 1 I-31100 Treviso Italy

## Abstract

**Background:**

An attenuation of the insulin-like growth factor 1 (IGF-1) signaling has been associated with elongation of the lifespan in simple metazoan organisms and in rodents. In humans, IGF-1 level has an age-related modulation with a lower concentration in the elderly, depending on hormonal and genetic factors affecting the IGF-1 receptor gene (*IGF-1R*).

**Methods:**

In an elderly population from North-eastern Italy (*n *= 668 subjects, age range 70–106 years) we investigated the *IGF-1R *polymorphism G3174A (*rs2229765*) and the plasma concentration of free IGF-1. Frequency distributions were compared using χ^2^-test "Goodness of Fit" test, and means were compared by one-way analysis of variance (ANOVA); multiple regression analysis was performed using JMP7 for SAS software (SAS Institute, USA). The limit of significance for genetic and biochemical comparison was set at α = 0.05.

**Results:**

Males showed an age-related increase in the A-allele of *rs2229765 *and a change in the plasma level of IGF-1, which dropped significantly after 85 years of age (85+ group). In the male 85+ group, A/A homozygous subjects had the lowest plasma IGF-1 level. We found no clear correlation between *rs2229765 *genotype and IGF-1 in the females.

**Conclusion:**

These findings confirm the importance of the *rs2229765 *minor allele as a genetic predisposing factor for longevity in Italy where a sex-specific pattern for IGF-1 attenuation with ageing was found.

## Background

Human longevity is the result of a complex interaction between genetics and environment (including lifestyle) and the contribution of each factor is difficult to quantify. Genetic components account for from 20 to 30% of lifespan, according to twin-based population studies [1,2].

In model organisms like *Caenorhabditis elegans*, *Drosophila *and the mouse, several genes involved in different basic cell pathways (stress response, DNA repair, microbial immunity and inflammation, metabolism and calorie restriction) have been shown to be related to longevity [3-5]. Genes belonging to evolution-conserved pathways, like the insulin/insulin like-growth factor 1 (IGF-1) network, are of particular interest in view of their key roles in cell physiology [6,7].

Insulin/IGF-1 signaling (IIS) triggers intracellular downstream transcription factors (for instance, belonging to the FOXO family) as the final step in a signal transduction pathway mediated by the homologous membrane receptors of insulin and IGF-1 [8]. Down-regulation of IIS has been associated with increased longevity, probably as a consequence of the decrease in cell growth and reduction in oxidative metabolism [9-11]. In humans the level of circulating IGF-1 is low in the elderly, but is higher in childhood than adulthood, probably as a consequence of different hormonal regulation. IGF-1 expression is sensitive to the growth hormone (GH) concentration and is also influenced by sex hormones [12-14].

Genetic variations in genes belonging to the IIS pathway have been explored in relation to longevity, dementia, metabolic diseases and cancer [15-18]. In the Italian population, a synonymous polymorphism (*rs2229765*) on the IGF-1 receptor gene (*IGF-1R*) [GenBank:NM_000875] consisting of a G to A transition at nucleotide 3174 leading to the amino acid change Glu->Glu at position 1043 (E1043E) [GenBank:NP_000876] was evaluated and the A-allele showed a positive association with aging and a negative influence on circulating levels of IGF-1 [19]. The same polymorphism has also been reported as predisposing factor for ischemic stroke in China [20].

We have collected a sample of elderly people (*n *= 668) from Treviso, Northeastern Italy, from 70 to 106 years old, homogeneous for ethnicity and geographical origin, and we have assessed their *rs2229765 *genotype and free IGF-1 plasma level.

## Methods

### Population sample recruitment

A comprehensive description of the study design has been reported [21]. Briefly, starting from a list of about 14,000 Treviso inhabitants over 70 years of age, a population sample was built up comprising three classes (70–79, 80–89, 90 years and over). The list from which the sample has been drawn comes from the population register. The final sample size was of 668 independent subjects (311 males and 357 females). The participants were evaluated from the biologic, clinical and socio-economic point of view, with a blood sample collection and a structured interview. The study protocol presenting the inclusion criteria, collecting procedure and questionnaire to be administered to all people involved in the project, was submitted to and approved by the Ethical Committee of the National Institute on Research and Care of the Elderly (INRCA, Italy). This protocol includes an informed, written consent to be obtained from the participant, or from a legally responsible relative in case of mentally impaired subjects, for clinical and genetic studies.

### Blood sampling and rs2229765 genotyping

Blood samples (about 30 ml) were collected by venipuncture; one part was used to isolate leukocytes for gDNA extraction, and the other was centrifuged at 2000 rpm for 10 minutes at 4°C (with sodium EDTA as anticoagulant) to separate the plasma fraction, which was then divided into aliquots and stored at -80°C until required. From the final population of 668 subjects, consent for blood collection was obtained from 590 Treviso inhabitants and, in all, 587 plasma samples were successfully prepared and stored for further analysis.

Genomic DNA was extracted from leukocytes using a semi-automated nucleic acid extractor (AB6100, Applera, USA) and stored at 4°C. To assess the *rs2229765 *genotype, a gDNA aliquot (about 50 ng) was amplified by polymerase chain reaction (PCR) using the following primers: forward 5'-tcttctccagtgtacgttcc-3', reverse 5'-ggaactttctctttaccacatg-3'. The resulting PCR product was digested by MnlI (New England Biolabs Inc, UK). The *rs2229765 *genotype was examined after loading the corresponding enzymatic digestions on a capillary electrophoresis unit with standard reference markers for instrumental lining-up (Agilent Technologies, USA).

From the available 590 gDNA samples, 510 samples gave unambiguous digestion result and were considered for subsequent analysis.

### Plasma IGF-1 assay

Plasma IGF-1 was assayed by a specific sandwich-type enzyme-linked immunosorbent assay (ELISA) (Diagnostic System Laboratories, Inc, USA), according to the manufacturer's instructions. The kit sensitivity was 10 ng/ml and the intra-assay %CV was <10%. From the assayed 587 plasma samples, 564 gave a IGF-1 value above the kit sensitivity limit and were considered for further analysis.

### Statistical analysis

Frequency distributions were compared using χ^2^-test "Goodness of Fit" test (Preacher, K. J. Calculation for the chi-square test: An interactive calculation tool for chi-square tests of goodness of fit and independence [Computer software]. ) and means were compared by one-way analysis of variance (ANOVA) followed by Tukey's or Dunnett's *post-hoc *test. Analyses were done using StatView program ver. 5.0. Multiple regression analysis was performed using JMP7 for SAS software (SAS Institute, USA). The limit of significance for genetic and biochemical comparison was set at α = 0.05.

## Results

### Age-dependent distribution of rs2229765

The demographic and the key clinical data of the population recruited in the TRELONG study are summarized in [Table 1]. The sample was representative of the general elderly Treviso population composition, ranging from 70 to over 100 years, with six individuals more than 100 years. Male-to-female ratio was 0.87. We divided the sample by five-year intervals as shown or by comparing two groups: the (70–85) group and the 85+ one, in an attempt to find longevity-related traits. We chose this cut-off value as several data suggest that after 85 years peculiar biochemical and metabolic features can be highlighted in comparison to the general population and also according to Italian population-specific survival curves [19,22,23]. This division gave roughly two thirds of the sample under 85 and one third over 85. Males and females were equally represented in the (70–85) group, while in the 85+ group women were over-represented (male-to-female ratio 0.67).

**Table 1:** Summary of the main demographic and clinical data of the TRELONG study.

Age bracket (no. of people)		No. of people (male:female)
70–74 (129)		61:68
75–79 (132)		72:60
80–84 (126)		64:62
85–89 (73)		29:44
90–94 (168)		74:94
95–99 (34)		10:24
100+ (6)		1:5
		
Total (668)		311:357

Measured variable	Unit	Mean ± SD

Age	years	84.0 ± 8.0
Platelets	N*10^3^/mm^3^	235.0 ± 75.0
White cells	N*10^3^/mm^3^	6.43 ± 1.70
Total cholesterol	mg/dl	214.0 ± 44.0
LDL cholesterol	mg/dl	136.0 ± 37.0
HDL cholesterol	mg/dl	56.0 ± 15.0
IgG	mg/dl	1125 ± 412
Body mass index (BMI)	Kg/m^2^	24.8 ± 4.1
Blood glucose	mg/dl	105 ± 33
DCI	-	2.3 ± 1.6
CCI	-	5.8 ± 2.0
		
Smoking status	Yes (278)	
	No (390)	

DCI: disease count index; CCI: Charlson comorbidity index; SD: standard deviation.

The entire study has been detailed elsewhere [21].

We first assessed the genotypic distribution of *rs2229765 *in our sample in five-year groups. No significant difference came to light (data not shown).

Then, we compared the *rs2229765 *genotypes in the (70–85) and the 85+ groups [Table 2]. The genotypic distributions respected Hardy-Weinberg equilibrium in both groups (data not shown) but there were no differences for either genotypic or allelic frequency in the whole sample. However, we found a sex-specific pattern as *rs2229765 *genotypic distribution in females under and over 85 was different from (70–85) males. Remarkably, at allelic level (70–85) males had a reduced A frequency (34.4%) in comparison to 85+ males (43.7%).

**Table 2:** *rs2229765 *genotypic and allelic frequencies according to age and sex.

	Genotype count (%)			Allele count (%)			
Age bracket (no. of people)	G/G	G/A	A/A	G	A	χ^2 ^p-value (genotypic distribution)	χ^2 ^p-value (allelic distribution)
70–85 (288)	102 (35.4)	147 (51.1)	39 (13.5)	351 (61.0)	225 (39.0)		
M (147)	59(40.0)	75 (51.0)	13 (9.0)	193 (65.6)	101 (34.4)		
F (141)	43 (30.5)	72 (51.0)	26 (18.5)^a^	158 (56.0)	124 (44.0)^b^	^a^p = **0.03**	**^b^p = 0.02**

85+ (222)	70 (31.5)	110 (49.5)	42 (19.0)	250 (56.3)	194 (43.7)		
M (89)	26 (29.2)	48 (53.9)	15 (16.8)	100 (56.2)	78 (43.7)^c^		**^c^p = 0.04**
F (133)	44 (33.1)	62 (46.6)	27 (20.4)^d^	150 (56.4)	116 (43.6)^e^	**^d^p = 0.02**	**^e^p = 0.02**

^a^: χ^2 ^p-value for the comparison of the genotype count distribution between males 70–85 and females 70–85; ^b^: χ^2 ^p-value for the comparison of the allele count distribution between males 70–85 and females 70–85; ^c^: χ^2 ^p-value for the comparison of the allele count distribution between males 70–85 and males 85+; ^d^: χ^2 ^p-value for the comparison of the genotype count distribution between males 70–85 and females 85+; ^e^: χ^2 ^p-value for the comparison of the allele count distribution between males 70–85 and females 85+.

M: men, F: women.

### Plasma IGF-1 concentration and relation between IGF-1 level and rs2229765 genotype

**Figure 1:**
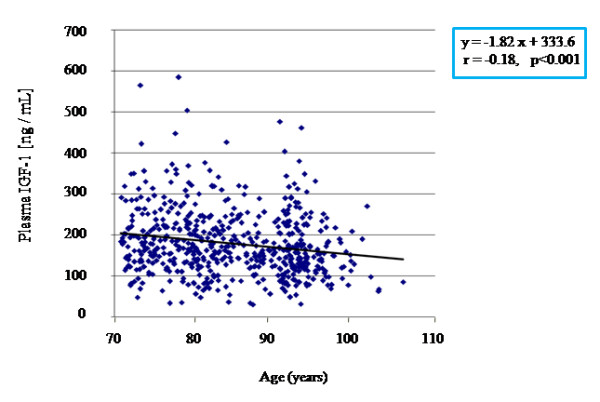
**Age-related reduction of circulating IGF-1**. Scatter-plot showing individual plasma IGF-1 levels measured by a dedicated ELISA kit, described in Methods. The superimposed line indicates the simple linear interpolation, whose equation and statistical significance are shown in the box on the graph. r: correlation coefficient.

We measured plasma IGF-1 concentrations in our whole sample and plotted the results according to age but regardless of sex [Figure 1]. The IGF-1 concentration decreased with age, and simple linear regression showed a significant negative correlation between age and IGF-1 level (r = -0.18, p < 0.001, sample size *n *= 564). To control for confounding factors, we then performed a multiple regression analysis using IGF-1 level as dependent variable and age, sex, body mass index (BMI), smoking, hypertension and blood glucose level as covariates [Table 3]. The correlation was confirmed with an even stronger significance level (p < 0.0001).

**Table 3:** Multivariate linear model using plasma IGF-1 levels as dependent variable.

	F	p-value
Age	16.3	**< 0.0001**
Sex	1.1	0.27
BMI	2.4	0.11
Smoking	3.4	0.06
Hypertension	2.2	0.22
Blood glucose	0.87	0.77
rs2229765*	1.8	0.19
sex (×) age**	2.3	0.11

Total sample size: *n *= 564. BMI: body mass index. *calculated as A- vs. A+ genotype; **sex and age interaction

The next step was to divide the population into five-year groups to check the longevity-related reduction in more detail [Table 4]. There was a significant decrease starting from 86 years in comparison to the 70–75 group, but the 91–95 group's mean IGF-1 level was not different from the first age bracket considered. However, the 96–100 group IGF-1 plasma level was significantly lower than the 70–75 group (a 24% reduction). This difference was even more evident in the extremely long-living subjects (40% less than the 70–75 group) but the small sample size did not give sufficient statistical power. This age-related IGF-1 reduction was evident by population stratification in ten-year groups, too [Table 4].

**Table 4:** Age-related IGF-1 plasma concentrations.

Age bracket	No. of people	IGF-1 [ng/mL] (mean ± SD)
*5 years*		
		
70 – 75	103	192 ± 80
76 – 80	119	197 ± 89
81 – 85	96	184 ± 77
86 – 90	73	159 ± 68**
91 – 95	138	171 ± 76
96 – 100	29	146 ± 51**
100+	6	117 ± 87

*10 years*		
		
70 – 79	195	199 ± 88
80 – 89	177	174 ± 70 #
90 – 99	186	168 ± 75 #
>99	6	132 ± 63

** **p < 0.01 **vs. (70–75) group and #p <**0.01 **vs. (70–79) group, one-way ANOVA followed by Dunnett's *post-hoc *test; SD: standard deviation, M: men; F: women.

To verify whether the age-dependent effect on IGF-1 was influenced by sex, we compared IGF-1 levels by sex and five-year intervals [Table 5]. We found that in males above 85 years IGF-1 decreased significantly while the mean plasma IGF-1 was the same in females under and over this cut-off value. This sex-specific effect was present also by ten-year intervals [Table 5].

**Table 5:** Sex-related decrease of IGF-1.

	Age bracket (no. of people)	plasma IGF-1 [ng/mL] (mean ± SD)
Males (*n *= 264)		
*5 years*	70–75 (49)	213 ± 88
	76–80 (66)	204 ± 76
	81–85 (50)	200 ± 81
	86–90 (32)	157 ± 65 **
	>90 (67)	159 ± 74 **
		
*10 years*	70–79 (99)	212 ± 83
	80–89 (85)	189 ± 76
	90–99 (79)	158 ± 72 §
	>99 (1)	63

Females (*n *= 300)		
*5 years*	70–75 (54)	175 ± 70
	76–80 (53)	189 ± 104
	81–85 (46)	164 ± 64
	86–90 (43)	161 ± 70
	>90 (104)	170 ± 73
		
*10 years*	70–79 (96)	185 ± 91
	80–89 (92)	159 ± 59
	90–99 (107)	173 ± 76
	>99 (5)	142 ± 86

Total subjects (*n *= 564)		
		
M (*n *= 264)		188 ± 80
F (*n *= 300)		172 ± 77#

**p < 0.01 vs. male (70–75) group; §p <**0.01 **vs. male (70–79) group; #**p < 0.05 **vs. total male subjects (*n *= 264), one-way ANOVA followed by Dunnett's *post-hoc *test. SD: standard deviation.

**Figure 2:**
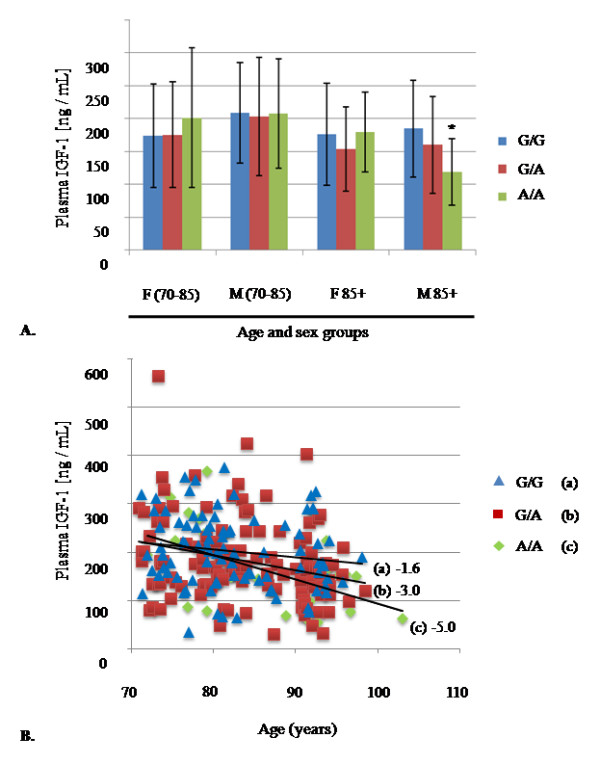
**Effect of sex and *rs2229765 *A-allele on IGF-1 circulating level**. **(A) **The sample was divided into two groups, older and younger than 85 years of age, then each group was divided according to sex. The distribution of IGF-1 concentrations in each group was stratified according to the *rs2229765 *genotype. The vertical bars represent the mean ± standard deviation (SD); *: **p < 0.05**, Tukey's *post-hoc *test vs. M 85+ G/G group; M: men, F: women **(B) **Scatter plot showing male *rs2229765 *genotype-specific linear regressions according to age. All males have been included. Regression slopes are reported next to each regression line.

To check for any correlation between *rs2229765 *genotype and mean IGF-1 level we stratified the sample by *rs2229765 *genotype. No significant change in IGF-1 concentration came to light (IGF-1 mean ± standard deviation: G/G 186 ± 78, *n *= 174; G/A 175 ± 81, *n *= 258; A/A 179 ± 83, *n *= 78; p > 0.05, one-way ANOVA). When we did a similar analysis in the (70–85) and 85+ groups we found a gender-specific pattern [Figure 2A]. In fact, in the men over 85 the homozygous A class had the lowest IGF-1 levels (119 ± 50 ng/mL, *n *= 15). This reduction was also present in the heterozygous G/A group (160 ± 74 ng/mL, *n *= 48) in comparison to the homozygous G/G class (185 ± 74 ng/mL, *n *= 26). However, the above reported difference was not robust (p = 0.048 for 85+ males A/A in comparison to G/G group in the same age bracket) and a chance effect cannot be excluded. We have also plotted the individual male IGF-1 levels for the whole population according to *rs2229765 *genotype and we have calculated the linear regression using age as independent variable. We noticed a genotype effect as the faster age-related decrease correlated with the A/A genotype while the G/G genotype decreased more slowly [Figure 2B]. In details, slope, r value and significance for each genotype were the following: (G/G) slope -1.6; r = 0.15; p = 0.15; (G/A) slope -3.0; r = 0.27; p = 0.002; (A/A) slope -5.0; r = 0.54; p = 0.03. A similar regression analysis was performed for females and the regression parameters were: (G/G) slope 0.13; r = 0.003.; p = 0.97; (G/A) slope -2.8; r = 0.29; p = 0.0007; (A/A) slope -1.6; r = 0.19, p = 0.09.

## Discussion

Human longevity is influenced by genetic and environmental factors. The present study addressed the question whether the genetic variability due to *rs2229765 *polymorphism of the *IGF-1R *plays a role in human longevity. We also sought a correlation between these genetic data and the circulating level of IGF-1, an important molecule in the general cell metabolism, whose reduction was found to be beneficial for longevity in model organisms [5-7].

We collected a homogeneous sample of people from Treviso (Italy), aged from 70 to 106 years (TRELONG Study) [21]. The sample was of particular interest to highlight the specific features of extremely long-living individuals (over 85 years) in comparison to those with a normal lifespan (between 70 and 85 years).

The polymorphism we assessed is a synonymous amino acidic substitution (E1043E) in the IGF-1R protein, whose functional significance is unclear. Genetic data about the *rs2229765 *did not indicate any major differences when the population was grouped in five-year age brackets or split in the two groups (70–85) and 85+, but there was an increase of the A-allele in the males over 85, suggesting a sex-specific effect on male longevity. The significance of this increase was not robust, (p = 0.04, χ^2^-test) probably because of the sample size of 85+ males. The (70–85) male group had a lower A-allele frequency than (70–85) females. This might be interpreted as a sex-specific effect but might also hide a chance or recruitment bias. A confirmatory study in an independent male population sample from Veneto Region is required to better clarify the above described limitations of the study. Our finding about *rs2229765 *A-allele was partially in agreement with a previous study in the Italian population by Bonafè *et al.*, which reported an increase of the *rs2229765 *A-allele in individuals over 85 years of age regardless of sex. This aspect can be explained considering that our study design was different, based on an elderly population sample, while Bonafè *et al. *considered a wider sample ranging from 20 to 100+ years [19]. Consequently, their increase of the A-allele in all the people over 85 years old might have depended on a larger difference between male A+ carriers in the two groups and does not exclude a sex-specific pattern. A gender-specific influence on longevity is a well-known aspect that can be partially explained at genetic level. Other polymorphisms show a gender-specific pattern in relation to longevity as well [24-26].

The second part of our study investigated plasma IGF-1 concentration, and a possible correlation between *rs2229765 *and IGF-1 circulating levels. We found a linear relationship between plasma IGF-1 and age using a simple and a multivariate regression model, confirming that IGF-1 continues to fall through life, however long. A substantial gender-specific effect was embedded in the general population analysis, as men and women contributed differently to the IGF-1 reduction: female IGF-1 in the (70–85) group was lower than in males but it did not drop further over time; in contrast, male IGF-1 was significantly lower in the 85+ group than the (70–85) group. Consequently, the drop in IGF-1 over time in our population was mainly due to a specific effect in males. This agrees with a reported sex-specific variation of IGF-1 level in model organisms, where it is influenced by sex hormones [27-29]. Bonafé *et al. *also reported a linear reduction of circulating free IGF-1 in the Italian population [19], in accordance with our data that magnified the effect for the elderly and found previously unreported sex-specific difference.

We found no difference in the entire sample as regards a correlation between IGF-1 plasma level and *rs2229765 *genotype. However, men showed a genotype-specific age-related decrease of plasma IGF-1 that declined faster when the subject was homozygous for the A-allele. In females this association was less evident, however the A-allele carriers had a faster plasma IGF-1 decline over age than the G/G subjects. This might suggest that in females *rs2229765 *impacts on circulating IGF-1 level, but this is masked by other genetic or environmental factors. This sex-specific relation was confirmed when we split our population at 85 years and divided men from women, as we observed that in the 85+ males the reduction of mean IGF-1 was unequally distributed among the genotypic classes G/G, G/A and A/A, the last group having the lowest IGF-1 concentration (119 ± 50 ng/mL), and the G/G genotype the highest (185 ± 74 ng/mL) that was comparable to the mean IGF-1 value of (70–85) men (205 ± 81 ng/mL). However, this correlation was statistically weak (p = 0.048, Tukey's test) and a chance effect should be taken into account. Recently, a genetic screening in Ashkenazi Jewish centenarians reported gender-specific modulations of circulating IGF-1 that was associated to *IGF-1R *genetic variability (including *rs2229765*), confirming that also this synonymous genetic alterations in the human *IGF-1R *might alter IGF signaling pathway and human longevity [30].

## Conclusion

This genetic and biochemical study in the Italian population confirmed and better detailed a genetic contribution to longevity coming from the A-allele of the *rs2229765 *polymorphism in the *IGF-1R *gene, that correlated with male longevity.

## Competing interests

The authors declare that they have no competing interests.

## Authors' contributions

DA drafted the paper and analyzed the results; MG drafted the paper, designed the study and performed the multivariate analysis; SB, LP, AV and MP performed the genotyping and ELISA assays; GBG, SDA, AZ collected the blood and plasma samples; GF critically read the manuscript.

## Pre-publication history

The pre-publication history for this paper can be accessed here:


